# Lost and Found: Return of the Inverted Repeat in the Legume Clade Defined by Its Absence

**DOI:** 10.1093/gbe/evz076

**Published:** 2019-04-08

**Authors:** In-Su Choi, Robert Jansen, Tracey Ruhlman

**Affiliations:** 1 Department of Integrative Biology, University of Texas at Austin; 2 Center of Excellence for Bionanoscience Research, King Abdulaziz University (KAU), Jeddah, Saudi Arabia

**Keywords:** *Medicago*, plastome evolution, alfalfa, homologous recombination, gene conversion, *accD*

## Abstract

The plant genome comprises a coevolving, integrated genetic system housed in three subcellular compartments: the nucleus, mitochondrion, and the plastid. The typical land plant plastid genome (plastome) comprises the sum of repeating units of 130–160 kb in length. The plastome inverted repeat (IR) divides each plastome monomer into large and small single copy regions, an architecture highly conserved across land plants. There have been varying degrees of expansion or contraction of the IR, and in a few distinct lineages, including the IR-lacking clade of papilionoid legumes, one copy of the IR has been lost. Completion of plastome sequencing and assembly for 19 *Medicago* species and *Trigonella foenum-graceum* and comparative analysis with other IR-lacking clade taxa revealed modest divergence with regard to structural organization overall. However, one clade contained unique variation suggesting an ancestor had experienced repeat-mediated changes in plastome structure. In *Medicago minima*, a novel IR of ∼9 kb was confirmed and the role of repeat-mediated, recombination-dependent replication in IR reemergence is discussed.

## Introduction

In the years since plastids were found to contain a DNA genome (plastome) distinct from that of the nucleus much has been learned about its gene content, organization and inheritance patterns. For the most part, the more than 2,900 annotated plastomes (NCBI, accessed December 11, 2018) representing all major lineages of photosynthetic eukaryotes conform to the expectation set by the first plastome data that became available using the tools of the time, such as electron microscopy, Southern analysis, Bac clones, and Sanger shotgun sequencing. The typical land plant plastome comprises the sum of repeating units of 130–160 kb in length found in all plastids, throughout all cells of the organism, regardless of developmental stage. The “unit genome” or “plastome monomer” is defined as a length of sequence that contains all of the genes and intergenic regions in one unit copy. Give or take a few commonly lost genes and/or introns the seed plant plastome monomer contains 115–118 unique genes with ∼17 of those duplicated in a long inverted repeat (IR). Sequences that are represented only once in the monomer are found in single copy (SC) regions that are unequally divided by the IR into the large and small single copy regions, LSC and SSC, respectively.

As early as 1976, Bedbrook and Bogorad had discovered that ∼15% of the corn (*Zea mays*) plastome was repeated in reverse orientation and contained the rRNA sequences (Bedbrook and Bogorad 1976). By 1979, Kolodner and Tewari published their finding that the plastomes of spinach (*Spinacia oleracea*), lettuce (*Lactuca sativa*), and corn contained a large IR and hypothesized that recombination reactions between the repeats could reverse the polarity of the intervening sequence (Kolodner and Tewari 1979). In different lineages, there have been varying degrees of expansion or contraction of the IR region, including or excluding genes and intergenic sequences from the IR, and this phenomenon accounts for most of the overall size variation among photosynthetic angiosperms (Ruhlman and Jansen 2014; Mower and Vickrey 2018). At its minimum, the IR contains a set of four ribosomal RNA and five transfer RNA sequences that is conserved in the green lineage, that is, from green algae through angiosperms, implying that this is the ancestral core of the plastome IR (Yamada 1991; Mower and Vickrey 2018). Sporadic expansion of the IR into both the LSC and the SSC regions throughout the evolution of land plants included eight additional genes, as seen in the plastome of *Amborella*, the basal member of the angiosperm clade (Zhu et al. 2016).

Despite near constancy among photosynthetic angiosperms, the IR has been highly reduced or completely eliminated from the plastome in disparate lineages. A recent report describes IR loss in one species of *Tahina* (Barrett et al. 2016), however this loss remains unconfirmed. The saguaro cactus, *Carnegiea gigantea*, lacks the IR and contains the smallest plastome sequenced to date among photosynthetic flowering plants (Sanderson et al. 2015). In Geraniaceae, IR variation has been elaborated to extreme degrees (Ruhlman and Jansen 2018). Although the IR is greatly expanded in the C2 clade of *Pelargonium* (the *P. transvaalense* IR duplicates ∼88 kb; Weng et al. 2017), *Monsonia* species sequenced thus far exhibit either a highly reduced IR (*M. speciosa*, 7,313 bp; Guisinger et al. 2011) or lack the canonical IR entirely (Ruhlman et al. 2017). Most plastomes from *Erodium* species lack the IR entirely except for those of the long branch clade (LBC) (Blazier et al. 2011) where a novel IR was identified. Three of the four LBC plastomes were completed and confirmed to contain IRs ranging from ∼25 to ∼47 kb that, among other coding sequences, include the ancestral core set of nine tRNA and rRNA genes. As to whether these *Erodium* IRs were retained within the LBC and lost in all other representatives of the genus or if they somehow reformed after a single loss on the branch leading to *Erodium* remains somewhat obscure. With the assumption that all other factors are equal, both trajectories are equally parsimonious requiring two steps: one loss followed by one gain, or two independent losses (Blazier et al. 2016).

It has been roughly 40 years since Kolodner and Tewari (1979) uncovered the earliest example of IR loss in pea (*Pisum sativum*). Subsequent restriction site and denaturation mapping identified the presence of the IR in mung bean (*Vigna radiata*), soy bean (*Glycine max*), and common bean (*Phaseolus vulgaris*) and confirmed its absence from pea and the closely related fava bean (*Vicia faba*) (Koller and Delius 1980; Palmer and Thompson 1981; Palmer et al. 1983). As information on the distribution of the IR among legumes accumulated, a single origin of IR loss within Fabaceae was suggested for the branch leading to a monophyletic subgroup of the Papilionoideae; three members of this clade, *Medicago sativa*, *Wisteria floribunda*, and *Trifolium subterraneum*, shared the same feature, whereas taxa outside did not (Palmer et al. 1987). Expanded sampling (Lavin et al. 1990) and the eventual shift to direct DNA sequencing has facilitated the exploration of many more legume plastomes from across the family and has supported the single origin hypothesis and confirmed the monophyly of the inverted repeat lacking clade (IRLC; Wojciechowski et al. 2000) within papilionoids (McMahon and Sanderson 2006; Schwarz et al. 2017). The complete plastome sequences for more than 100 Fabaceae are currently available, with ∼75% representing the papilionoid legumes, 40 of which are IRLC taxa (accessed October 9, 2018). Both molecular phylogenies and completed plastomes robustly support the branch leading to the IRLC as the unique origin of IR loss in Fabaceae.

The genus *Medicago* contains about 87 species (Small 2011) and belongs to tribe Trifolieae, which is nested firmly within the IRLC (Cardoso et al. 2015; Legume Phylogeny Working Group 2017). Alfalfa (*Medicago sativa* subsp. *sativa*) is the most important forage crop in the world, ranking fourth among all crops grown in the United States (Small 2011). It is an excellent source of animal nutrition as it is highly digestible and rich in vitamins, minerals, and proteins, including six essential amino acids among them sulfur-containing methionine. Alfalfa generates 7 billion dollars annually in the United States as a forage crop and its seed and sprouts are estimated to generate $450 million annually making it a significant target for genomic resource development. In addition to alfalfa, the genus contains the important research species *Medicagotruncatula*, a diploid whose high reproductive rate and amenability to genetic manipulations have made it an indispensible model system for legume biologists and of interest to plant biologists in general. With respect to plastomes, *Medicago* does not appear highly rearranged relative to congeners, unlike *Trifolium* another genus in the Trifolieae (Cai et al. 2008; Sveinsson and Cronk 2016). Previous studies of plastome characteristics in the genus are limited and there are currently a total of four species represented in the NCBI database.

To explore plastome characteristics within *Medicago* and examine potential markers for breeding experiments 19 plastomes representing all the major clades in the genus *Medicago* along with one species of *Trigonella*, were sequenced, assembled and annotated. For the most part *Medicago* plastomes are syntenic. Unexpectedly, a novel IR was identified in one clade that reaches up to ∼9 kb in one species and includes a portion of the ancestral IR core.

## Materials and Methods

### Taxon Selection, Acquisition, Cultivation, and Harvest

Taxa were selected to represent all the major clades in the *Medicago* phylogeny (supplementary table S1, Supplementary Material online) (Maureira-Butler et al. 2008) and included 19 species plus *Trigonella foenum-graceum*. Seed of all accessions was acquired from the United States Department of Agriculture Germplasm Resources Information Network (GRIN). Seeds were germinated in the UT greenhouse and emergent leaves from a single plant from each accession were flash frozen in liquid N_2_ and stored at −80 °C. A representative of each accession was harvested at maturity for voucher preparation. Vouchers were deposited in the herbarium at the University of Texas at Austin (TEX-LL) and voucher accession numbers are in supplementary table S1, Supplementary Material online.

### DNA Sequencing, Assembly, and Annotation

Genomic DNA isolation by the cetyl trimethylammonium bromide method followed Doyle and Doyle (1987) with modifications. Cetyl trimethylammonium bromide buffer was augmented with 3% PVP and 3% beta-mercaptoethanol (Sigma, St. Louis MO). Organic phase separation was repeated until the aqueous fraction was clear. DNA pellets were resuspended in ∼200-μl DNase-free water. Following treatment with RNase A (ThermoScientific, Lafayette, CO), samples were again subjected to phase separation with chloroform. DNA was recovered by ethanol precipitation, resuspended in DNase-free water, and stored at −20 °C. Samples were shipped to Beijing Genomics Institute (BGI; Hong Kong). Sample quality was assessed by BGI and qualified samples were sheared to produce a fragment library with ∼250-bp inserts. For each taxon a minimum of 20 million 150-bp paired-end reads were collected using Illumina HiSeq X-ten platform (Illumina, San Diego, CA).

The total of quality filtered reads were trimmed in Geneious v. 7.1.9 (https://www.geneious.com) (Kearse et al. 2012) using the default parameters. Approximately 10% of trimmed reads were assembled de novo in Geneious with low sensitivity. Complete and partial assemblages for organelle genomes and nuclear ribosomal DNA region were returned. The plastome was recovered in one or two contigs for each taxon. In cases where two contigs were returned the reiteration method described by Wang and Messing (2011) was employed. Briefly, the procedure of read mapping and assembly of mapped reads was reiterated until a single plastid contig was obtained using each of the de novo assembled plastid contigs as a reference. The unit genome assemblies were completed by trimming overlapping regions of each plastome contig. Each completed plastome assembly was refined and confirmed by mapping of total quality filtered and trimmed reads in Geneious.

The completed plastomes were annotated using GeSeq (Tillich et al. 2017) with MPI-MP chloroplast references. All tRNAs were confirmed by tRNAscan-SE v2.0 (Lowe and Chan 2016). For confirmation, all annotations were compared with previously published plastomes of Fabaceae downloaded from NCBI (https://www.ncbi.nlm.nih.gov/genome/organelle/) and exon boundaries were manually corrected in Geneious.

### Phylogenetic Inference

Plastome sequences of 28 taxa were included in phylogenetic analyses. In addition to the 20 newly sequenced plastomes (see supplementary table S1, Supplementary Material online, for GRIN plant identification and voucher numbers), six accessions from the IRLC (*W.floribunda* [NC_027677], *Astragalus mongholicus* [NC_029828], *Cicer arietinum* [NC_011163], *Trifolium aureum* [NC_024035], *Vicia faba* [KF042344], and *M.truncatula* [JX512022]) and two robinioid species (*Robinia pseudoacacia* [KJ468102] and *Lotus japonicus* [NC_002694]) representing the sister group to the IRLC (Legume Phylogeny Working Group 2017) were selected. The coding regions of 69 genes shared across taxa (supplementary table S2, Supplementary Material online) were extracted from each plastome and concatenated. Sequences were aligned with MAFFT v.7.017 (Katoh and Standley 2013), implemented in Geneious, using the default settings. A maximum likelihood analysis was performed with RAxML v.8 (Stamatakis 2014), as implemented in CIPRES Science Gateway (Miller et al. 2010), using GTRCAT as model and 1,000 bootstrap replications.

### Repeat Content Estimate and Inversion Inference

Repeat content was calculated for all sequenced plastomes; tandem repeats were characterized by Tandem Repeats Finder version 4.09 (Benson 1999) with default settings. Dispersed repeats were identified by using each plastome as both subject and query in BlastN analysis (v2.8.0+;) with a word size of 7 and an *e*-value of 1e-6 to detect repeats ≥30 bp (Guo et al. 2016). Further refinement to exclude nested or overlapping repeats was carried out to avoid overestimation of repeat content. Specific repeat sequences longer than 250 bp were investigated in *Medicagosuffruticosa* and *Medicagolupulina* by polymerase chain reaction (PCR) amplification and Sanger sequences. Primer sequences are presented in supplementary table S3, Supplementary Material online.

The arrangement of locally colinear blocks among the 20 newly sequenced plastomes was estimated using progressiveMauve 2.3.1 (Darling et al. 2010) in Geneious. Inversions were identified in *Medicago* relative to *W.floribunda*, an early diverging IRLC taxon.

### Alignment of *accD* Coding Regions

The *accD* coding sequence of *M.truncatula* (JX512022) was aligned with those of the 20 taxa sequenced in this study using MAFFT and the translation align function in Geneious. Translated AccD sequences were aligned using the Geneious Aligner with default settings. The protein alignment was annotated for active site residues in the carboxyterminus (Lee et al. 2004; Gurdon and Maliga 2014).

### Confirmation of a Novel IR in *Medicago minima*

The presence of a large IR detected in *M. minima* was confirmed by PCR and Sanger sequencing of the boundary regions. Oligonucleotide primers were designed using Primer3 (Untergasser et al. 2012) and are given in supplementary table S3, Supplementary Material online, along with target sites and expected amplification products.

Mapping of all quality filtered and trimmed reads to the assembly of the *M. minima* unit genome was carried out in Geneious as described above. Reads were mapped against the entire monomer sequence as well as to a version from with one copy of the IR removed.

## Results

### Plastome Sequencing and General Characteristics

**Fig. 1.**
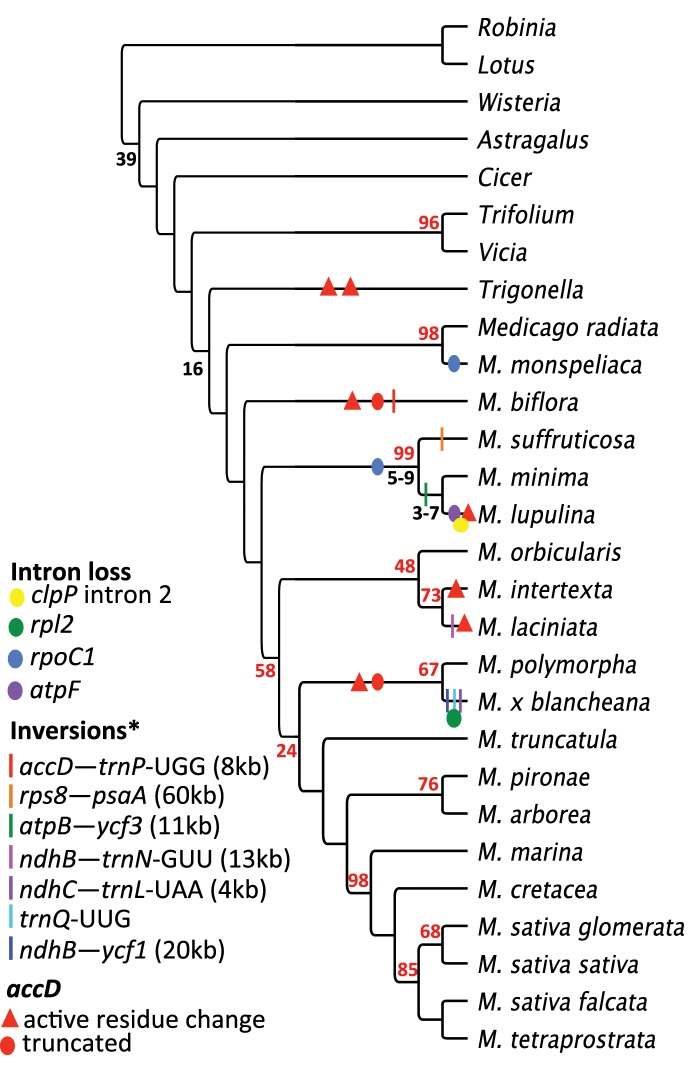
—Phylogenetic relationships and distribution of plastome structural variation. Shared coding sequences (69; see supplementary table S2, Supplementary Material online) were extracted from newly sequenced *Medicago* and *Trigonella* plastomes and combined with *M. truncatula* and seven additional legume taxa for construction of a phylogenetic tree. Structural features are plotted on the branches of the cladogram and indicated in the key (inset) and inversions are relative to *Wisteria floribunda* (asterisk). At the nodes, black numerals indicate divergence estimates in millions of years (*Medicago*, de Sousa et al. 2016; IRLC, Lavin et al. 2005) and bootstrap values <100 are indicated by red numerals. Active site residue changes in the Acetyl-coenzyme A carboxylase carboxyl transferase subunit beta polypeptide are diagrammed in supplementary figure S1, Supplementary Material online.

In all, plastome sequences were completed for 20 taxa, 19 *Medicago* and one outgroup, *Trigonella foenum-graceum* (fig. 1 and table 1). The total number of quality filtered reads and average depth of plastome coverage are reported in table 1. The *Medicago* plastomes lacking a large IR ranged in size from ∼121 to ∼126 kb and all plastomes were fairly consistent with regard to GC content at 33–34% (table 1). GenBank accession numbers for all newly sequenced taxa are reported in supplementary table S1, Supplementary Material online.

**Table 1:** Sequencing and Plastome Statistics

Taxon	Quality Filtered Reads	Average Depth of Coverage	Size (kb)	Repeat Content^a^ (%)	GC (%)
*Trigonella foenum-graceum*	61,540,540	5,353	125,645	5.28	33.9
*Medicago radiata*	48,875,990	4,410	124,564	3.63	33.9
*M. monspeliaca*	49,048,384	5,664	121,313	5.35	33.9
*M. biflora*	49,142,116	3,106	121,957	4.10	33.9
*M. suffruticosa*	48,805,382	4,109	126,394	6.73	34.1
*M. lupulina*	69,724,052	6,717	122,770	6.68^b^	34.0^b^
*M. minima*	48,948,214	8,267	132,296	5.25^b^	34.2^b^
*M. orbicularis*	47,374,178	7,160	125,015	4.04	33.8
*M. intertexta*	49,054,258	4,530	125,621	5.21	33.9
*M. laciniata*	48,475,902	5,337	123,500	3.99	34.0
*M. polymorpha*	48,335,998	5,041	124,066	4.32	34.1
*M.* x *blancheana*	48,584,452	5,889	123,387	7.03	34.5
*M. pironae*	68,530,854	4,394	123,712	3.70	33.9
*M. arborea*	49,150,052	5,689	124,273	4.48	34.1
*M. marina*	48,979,352	3,321	124,182	4.08	33.8
*M. cretacea*	66,805,720	5,504	126,021	5.17	33.7
*M. sativa* subsp. *glomerata*	68,694,194	4,657	125,889	5.60	33.9
*M. sativa* subsp. *sativa*	76,967,206	5,889	125,330	4.39	33.9
*M. sativa* subsp. *falcata*	65,459,364	5,937	126,016	5.23	33.8
*M. tetraprostrata*	73,561,680	6,134	126,778	6.05	33.7

a Tandem and dispersed repeats ≥30 bp.

b GC% and repeat content of IR plastomes were calculated using only one IR copy.

**Fig. 2.**
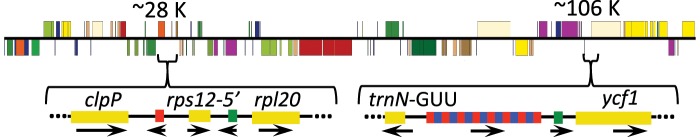
—Plastome repeats in *Medicago suffruticosa*. Repeats identified in *M. suffruticosa* suggests repeat-mediated phenomena in a common ancestor of the clade that includes this species and *Medicago minima* may have been involved in initiation of the novel IR in *Medicago minima*. The upper portion of the diagram shows the *M. suffruticosa* unit genome map, below are the regions of interest. Thick black lines represent double stranded DNA. Colored boxes in the plastome map (above) represent gene sequences and colored by functional groups. Values above the map indicate the loci of regions of interest (below). Bracketed regions of interest contain genes (yellow) and repeats (red, blue, and green). Arrows indicate the strand for each coding region and repeat.

Among most *Medicago* species the abundance of repeats greater than 30 bp (range 30–834 bp) was below 6% however three taxa, *M. suffruticosa*, *M. lupulina*, and *M.* x *blancheana* have accumulated up to ∼7% repetitive sequence (table 1). *Medicago* x *blancheana* also contains a tandem repeat that duplicates the *trnQ*-*UUG* gene. Repeat sequences longer than 250 bp were mainly distributed in intergenic regions and around specific genes in *M. suffruticosa* and *M. lupulina* (*clpP*, *rps12*, *trnN*-GUU, *ycf1*, *rpl20*, *trnV*-GAC, and *rrn16*) and were confirmed by PCRs and Sanger sequencing (supplementary table S3, Supplementary Material online). *Medicago suffruticosa*, which had the second greatest accumulation of repeated sequence (6.73%), had a series of tandem repeats in the region between *trnN*-GUU and *ycf1* (coordinates 106,467–107,072); the 79 bp sequence is repeated in full seven times at 100% identity (red/blue block in fig. 2). The first 30 bp of the 79-bp repeat (red block) is also present as a single inverted sequence of high identity (81.3%) at coordinate 30,301, between the *rps12-5*′ and *clpP* genes of the *M. suffruticosa* plastome. Downstream of the tandem array lies a 29-bp sequence (green block; coordinate 107,134) that is present between the *rps12-5*′ and *rpl20* genes (coordinate 31,225), also in inverted orientation with 83.3% identity. The 29-bp sequence at the upstream position (∼coordinate 35K in *M. suffruticosa*) is conserved across the genus in both position (∼coordinate 66K) and nucleotide identity (>92%), however it is in opposite orientation relative to *M. suffruticosa* as this plastome contains an inversion (fig. 1) that reversed the polarity of the segment that contains the upstream repeat (∼coordinate 28K; fig. 2).

Apart from repeats, other noteworthy genomic changes were plotted on a phylogeny based on shared genes across all included taxa (fig. 1 and supplementary table S2, Supplementary Material online). In terms of overall structure, ProgressiveMauve identified further variation in the arrangement of locally colinear blocks relative to the outgroup *W. floribunda*. A total of seven inversions were identified and plotted on the cladogram in figure 1 along with several instances of intron loss and *accD* divergence (bars, dots [see key], and triangles, respectively). Including *M. suffruticosa*, six inversions are unique to a single species, whereas one is shared by *M. lupulina* and *M. minima*. In common with *M. truncatula*, the sequence of *accD* was interrupted by complex tandem repeats that varied across taxa. In three cases, the *accD* sequence was highly divergent and truncated relative to close congeners and may be nonfunctional (red oval fig. 1 and supplementary fig. S1, Supplementary Material online). Nucleotide alignment of the gene and amino acid sequences showed that most conserved residues are retained in the carboxyterminus of *Medicago accD* (supplementary fig. S1, Supplementary Material online).

### A Novel IR Uncovered in *Medicago*

**Fig. 3.**
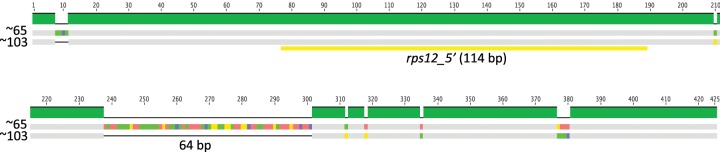
—Small IR in *Medicago lupulina*. A small IR was identified in *Medicago lupulina* situated in the same genomic location as the expanded IR of *Medicago minima* and includes the coding region of *rps12_5*′ (yellow bar)*.* The IR comprises 425 bp (aligned length) and includes an indel of 64 bp. Numerals at the left indicate the genomic position of the repeat copies (approximate coordinates) and numerals above indicate nucleotide positions within the repeat alignment. Mismatches are indicated by colored blocks and identical bases are gray. Mean pairwise identity over all pairs in each alignment column is indicated by the histogram: green 100%.

Sequence assembly of the *M. minima* plastome using a range of parameters (see Materials and Methods) suggested the presence of a large inverted repeat (9,248 bp: coordinates 64,387–73,634 and 104,036–113,283) that contains 7 coding sequences compared with ∼17 in the typical IR of angiosperms. Duplicated sequences have been derived from both affected regions of the plastome and include the ribosomal RNA genes *rrn23*, *rrn4.5*, and *rrn5*, along with two tRNA genes typically situated upstream of *ycf1* (retained genes of IR_B_ across the IRLC; coordinate 105,512 in *M. radiata*), and two protein coding genes (*clpP* and *rps12-5*′), typically situated downstream of *rpl20* (coordinate 67,107 in *M. radiata*). Assembly of plastome reads from *M. lupulina*, sister to *M. minima*, also suggested the presence of smaller inverted repeat (aligned length of 425 bp) that contains a single coding sequence, the gene encoding *rps12-5*′ (coordinates 64,563–64,987 and 103,362–103,006). In *M. lupulina*, the duplicated, inverted sequence is interrupted by a gap (64 bp) in the downstream repeat (fig. 3). This 64-bp intergenic sequence was identified in the upstream location (∼coordinate 65 kb) across nine of the included *Medicago* species with one to four nucleotide differences. Excluding the region absent from the downstream repeat results in 96.7% identity over 361 bp.

**Fig. 4.**
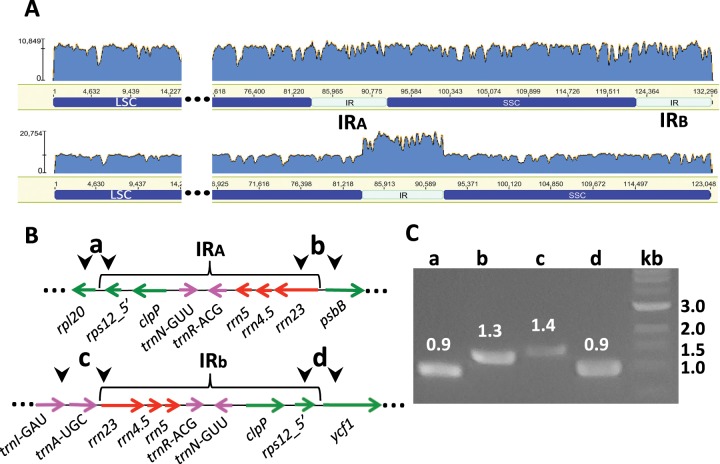
—Confirmation of IR extent in *Medicago minima*. Plastome reads were mapped (*A*) to the assembled *Medicago minima* plastome containing both (upper) or a SC (lower) of the large IR. The scale at the left reports the depth of reads, which is indicated graphically by the blue histogram. To amplify IR boundaries (*B*), oligonucleotide primers (arrowheads) were selected to anneal inside and outside the repeated region. Brackets enclose IR_A_ and IR_B_, colored arrows indicate coding sequences. Drawing is not to scale. All products (*C*) were evaluated by electrophoresis and submitted for Sanger sequencing. Numeric values in white indicate product sizes. The lowercase letters in (*B*) correspond to the lanes in (*C*). kb, kilobases.

Read mapping and PCR were performed to confirm an assembly for *M. minima* that includes the novel ∼9 kb IR. All plastome reads for *M. minima* were mapped to the assembly in Geneious. A graphical depiction of the mapping result (fig. 4*A*) demonstrates even distribution of reads over the assembly when both copies of the repeat are included (above) and ∼2-fold higher depth of coverage over the repeated region, relative to SC regions) when one repeat copy is excluded (lower). Amplification primers designed to bridge all four IR/SC boundaries (fig. 4*B* and *C* and supplementary table S3, Supplementary Material online) produced fragments of the expected size in each case (fig. 4*C*; a, 0.9 kb; b, 1.3 kb; c, 1.4 kb; and d, 0.9 kb) and Sanger sequencing confirmed the boundaries.

**Fig. 5.**
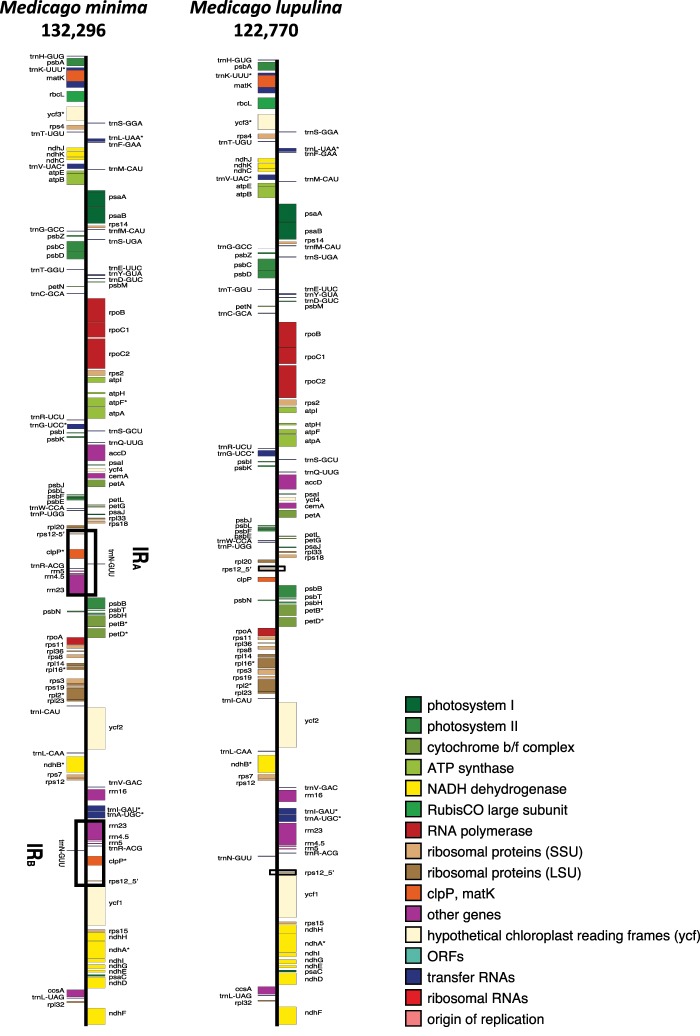
—Unit genome maps for *Medicago lupulina* and *Medicago minima.* The completed, annotated plastome assemblies were submitted to OGdraw (Lohse et al. 2013; https://chlorobox.mpimp-golm.mpg.de/OGDraw.html) to generate annotated maps for visualization of the unit genome. Unit genome size in kilobases is given below each taxon name. The IR in *Medicago minima* is contained in open black boxes as is the small IR in *Medicago lupulina*. The thick black line represents the plastome monomer and the filled, labeled boxes represent coding regions. Asterisks indicate genes containing introns. The functional group of each gene is indicated by color in the legend (lower right).

Linear maps depicting the *M. minima* and *M. lupulina* are presented in figure 5.

## Discussion

Despite all that has been learned about the structure and function of plastomes, there remain many misconceptions obfuscating the mechanisms of plastome replication and repair, and concomitantly the drivers of plastome evolution. Repeat-mediated changes in plastome architecture that neglect interactions between plastome copies, present as circular and linear multimers and complex linear-branched forms, limit illuminating hypotheses. Recombination between homologous and/or homeologous sequences within and between unit genome copies can yield various arrangements including inversions, duplications and deletions of sequence in one or both of the interacting units (Day and Madesis 2007; Maréchal and Brisson 2010; Zampini et al. 2017). A consideration of plastome structural/gene order evolution, such as expansion, contraction, and the loss or gain of the plastome IR in some lineages, must consider both intra and intermolecular interactions.

Although once thought to occur through intramolecular recombination between the two copies that make up the IR, inversion of the SC regions requires that the recombination reaction to initiate replication occur between IR sequences present in different units of the plastome monomer arranged in head-to-tail linear concatamers (Oldenburg and Bendich 2004; Maréchal and Brisson 2010). Likewise, DNA repair mechanisms that rely on homologous recombination (HR) may utilize loci within a single unit (between repeated sequences), or between sequences within two different units. Replication in IRLC plastomes and other taxa that lack the plastid IR must necessarily initiate at a homologous site in another plastome copy.

**Fig. 6.**
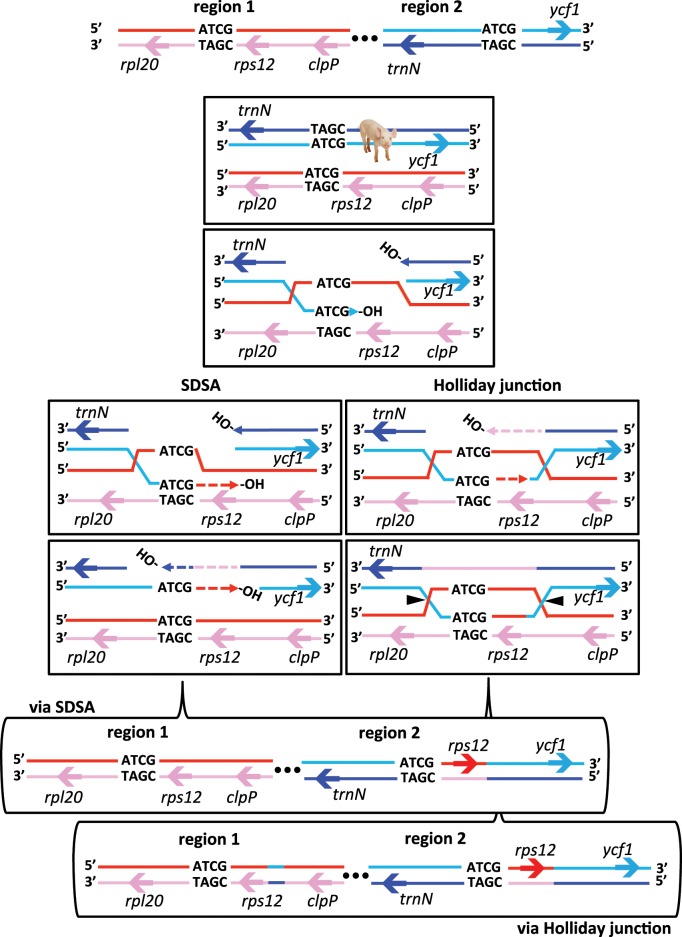
—Recombination based models for initiation of IR growth in the *Medicago minima* clade. Establishment of the novel IR in the *Medicago minima* clade could have occurred either by SDSA or through HJ formation. At the top are the two regions of interest involved in IR formation. The ellipsis indicates that the two involved regions are within the same plastid nucleoid, but may or may not be physically connected. The letters “ATCG” represent a direct repeat and do not refer to a specific sequence or sequence length. Solid lines represent intact DNA and dashed lines indicate newly synthesized strands. Following a double-ended double strand break (plastome integrity gap or PIG) in the recipient duplex (blue/teal) both 5′ ends are resected to yield 3′ overhangs that can invade the intact donor duplex (red/pink) at a homologous/homeologous site (ATCG) forming a D-loop. In this diagram, the light blue recipient strand has invaded the donor strand. In SDSA (left), extension beyond the site of damage triggers disassociation of the donor and recipient duplexes. DNA synthesis fills in gaps with the involvement of mismatch repair resulting in gene conversion without crossing over. Formation of a HJ (right) permits DNA synthesis beyond the original break site. The displaced donor strand (red) templates DNA synthesis anneals to the other recipient strand (blue). Extension is followed by ligation of the free ends resulting in two HJs. Provided HJ resolvase (black arrow heads) acts on the indicated sites (side cuts), gene conversion without crossing over occurs. Resolution of the junctions in the other orientation (top/bottom cuts) will result in gene conversion with a crossover, however the data support side cut resolution.

HR is an integral part of DNA maintenance in plastomes and is employed by both replication and repair pathways (Day and Madesis 2007; Maréchal and Brisson 2010; Zampini et al. 2017). The regeneration of an IR in *M. minima* may have proceeded through HR between forward repeats in the regions that house this feature on different copies of the unit genome. Repair of double strand breaks that templates DNA synthesis through HR between imperfect, nonallelic repeats is a plausible mechanism (Maréchal et al. 2009; Kwon et al. 2010). Two distinct double strand break repair pathways may be proposed to initiate the novel IR identified in *M. minima*; double strand break repair via Holliday junction (HJ) formation and resolution and synthesis-dependent strand annealing (SDSA; right and left, respectively; fig. 6). In figure 6, the “ATCG” represents a repeat of no defined length and is not meant to imply complete identity between interacting repeats during HR. The actual sequences involved in the reactions that led to repeat accumulation in *M. suffruticosa* (fig. 2) and perhaps eventually to IR establishment in *M. minima*, may no longer be present in their plastomes and there is no way to validate or refute plausible mechanistic hypotheses based on the current data. Considering the positions of the repeats identified in *M. suffruticosa* (fig. 2), the novel IR in *M. minima*, and the presence of a quasi-IR in *M. lupulina*, it is reasonable to hypothesize that a unique perturbation occurred in the ancestor of this clade. The common ancestor likely had the same gene order as the *M*. *lupulina*/*M*. *minima* clade, as the rearrangement that inverted the upstream repeats (∼coordinate 28K in *M*. *suffruticosa*; figs. 1 and 2) occurred after the divergence of *M*. *suffruticosa* (de Sousa et al. 2016). The tandem array in *M*. *suffruticosa* (∼coordinate 106K) could represent an artifact of DNA replication or repair gone awry. At the time of divergence (∼5–9 Myr) (de Sousa et al. 2016) a syndrome of repeat accumulation may have been shared between the two lineages and subsequent resolution has followed different paths. It is possible that the inversion that changed the polarity of the repeats in question halted a trajectory that relied on direct repeats in *M. suffruticosa* but continued into the *M*. *lupulina*/*M*. *minima* clade. Alternatively, the repeat accumulation in *M. suffruticosa* may have been exclusive to that lineage arising after the divergence.

There seems an obvious connection between the quasi-IR of *M. lupulina* and the 9-kb IR of *M. minima*. It is likely that the seminal event that gave rise to the novel IR was shared between both species. Duplication, relocation and inversion of the sequence including *rps12-5*′ was likely an early stage of that event as this feature is shared by both. In figure 6, the repeat (ATCG) involved with nonallelic pairing for HR-mediated DNA repair is placed just upstream of *rps12-5*′. Longer (or shorter) stretches of sequence could have been included in the initial event that were subsequently expunged or expanded in *M*. *lupulina* and *M*. *minima*, respectively. The continued expansion of the novel IR in *M*. *minima* proceeded to include three ribosomal RNA and two tRNA genes. The repeat (ATCG) diagrammed in figure 6 could correspond to the “green” repeat in figure 2. The copies of this 29-bp repeat in *M*. *suffruticosa* share 83.3% sequence identity and are positioned in such a way that, if present in the ancestor of *M*. *lupulina*/*M*. *minima*, could facilitate a mechanism producing the IR structures observed in the clade (figs. 1 and 6). Repeat-mediated inversion is common in plastomes, likely proceeding through HR dependent mechanisms acting on imperfect repeats or simple sequences such as mono- or dinucleotide runs that accumulate in noncoding regions (as in *M*. *truncatula*) (Gurdon and Maliga 2014). The association of poly A(T) tracts and IR expansion has been suggested previously (Goulding et al. 1996; Lee et al. 2007; Wang et al. 2008; Dugas et al. 2015). It is possible that deeper sampling in this part of the *Medicago* phylogeny will illuminate the phenomena that gave rise to the >9-kb IR of *M. minima*.

Although the plastome IR has persisted in most lineages of photosynthetic angiosperms since their divergence at least 140–150 Ma (APG IV) (Chase et al. 2016), there are examples of its loss from contemporary plastomes. Perhaps the most widely recognized loss occurred ∼39 Ma (Lavin et al. 2005) within the papilionoid legumes and defines the IRLC (Wojciechowski et al. 2000). Other unambiguous losses have been identified among the species of three Geraniaceae genera (*Monsonia*, *Geranium*, and *Erodium*) (Guisinger et al. 2011; Blazier et al. 2016; Ruhlman et al. 2017) and also in *C.gigantea* (saguaro cactus) (Sanderson et al. 2015). Because just a single *Carnegiea* plastome has been sequenced within cactus family (Cactaceae) thus far it is unknown how extensive or ancient the loss in this group may be. At one time the loss in *Erodium* was placed at the base of the genus, dating back 18 Ma (Fiz et al. 2008; Guisinger et al. 2011), however recent findings revealed the presence of a large, novel IR in members of the LBC. If indeed the IR in the LBC was regained then the placement on the basal branch is logical. Equally plausible by a parsimony argument is that two independent IR losses occurred within clades containing *Erodium* plastome types 1 and 3 and type 2, which carry divergence time estimates of 8 and 15 Ma, respectively (Fiz et al. 2008; Blazier et al. 2016).

Unlike the case in *Erodium*, the presence of a novel IR in *M. minima* leaves little doubt regarding retention versus acquisition. Nested within the IRLC, a group with many sequenced plastomes, and within *Medicago*, which is now represented by more than 20 plastome sequences from across the genus, *M. minima* is the only taxon identified to contain a structure similar to the canonical plastome IR. Clearly the novel IR arose within the *M*. *lupulina*/*M*. *minima* clade (3–7 Ma) (de Sousa et al. 2016), or on the branch leading to *M*. *suffruticosa*/*M*. *lupulina*/*M*. *minima* (5–9 Ma) (de Sousa et al. 2016) culminating in *M. minima* with the boundary expansion to include typical IR genes encoding ribosomal and transfer RNA. Given that IR boundary migration is a dynamic process that varies its length and content even among closely related taxa (Goulding et al. 1996; Ruhlman and Jansen 2014; Zhu et al. 2016) it is entirely possible that the >9-kb IR in *M. minima* will continue to expand and eventually include more of the adjacent canonical IR genes.

Each new discovery of IR loss, or gain, ignites the speculation: Why IR? Although a number of hypotheses have been suggested, some tested, a satisfying explanation of the nature and necessity of the plastome IR remains elusive. The presence of the IR per se is not essential for plastid function, as several groups of fully autotrophic flowering plants along with several gymnosperm lineages have dispensed with it to no detriment. The suggestion that the IR was required to stabilize plastomes was a reasonable one as early investigations correlated plastomes lacking the IR with the observation of more frequent genomic rearrangement (Palmer and Thompson 1982). Sequencing of the rearranged plastomes of *Erodium texanum* and *Carnegieagigantea* could be taken as support for this hypothesis. However, there are many examples of highly rearranged plastomes with a canonical IR in Campanulaceae (Haberle et al. 2008; Knox 2014), Ericaceae (Fajardo et al. 2013; Martínez-Alberola et al. 2013), Geraniaceae (Chumley et al. 2006; Guisinger et al. 2011; Weng et al. 2014), and Passifloraceae (Rabah et al. 2019).

Replication initiation via the IR embedded origins could provide an explanation for its broad presence and persistence, yet plastomes that contain, as well as lack, the large repeat produce replication intermediates originating in sites outside of the IR (i.e., *M. truncatula*; Shaver et al. 2006). This indicates that plastome replication may be initiated at other sites and may be independent of the characterized replication origins; both are dispensable in *Nicotianatabacum* (Scharff and Koop 2007). Furthermore, replication initiation from within the IR likely proceeds through recombination between IR copies in different unit genomes, despite previous notions of a circular and/or monomeric molecule (Kunnimalaiyaan and Nielsen 1997; Oldenburg and Bendich 2004; Day and Madesis 2007; Scharff and Koop 2007; Zampini et al. 2017). Consider that recombination-dependent replication can only produce both SC isoforms in equimolar proportions if all, or nearly all, replication initiates via recombination between IR copies in different plastome units.

Several studies have examined the effect of IR inclusion or exclusion on nucleotide substitution rates of protein coding sequences. The highly iterative nature of plant plastomes employs gene conversion to maintain the identity of the many unit copies (Birky and Walsh 1992; Khakhlova and Bock 2006). It stands to reason that a length of sequence that is duplicated should undergo gene conversion, which requires recombination, at twice the rate of SC sequences and findings in several different groups have generally supported a reduction in synonymous substitutions for IR genes relative to those in the SC regions, although not always by a factor of two. Regardless of the specific sites or species selected for the comparison depression of the synonymous substitution rate based on location in the IR versus SC regions was reported for typical plastomes (Wolfe et al. 1987; Gaut et al. 1993; Maier et al. 1995; Perry and Wolfe 2002; Yamane et al. 2006; Kim et al. 2009; Yi and Kim 2012; Yi et al. 2012; Zhu et al. 2016).

Despite their IR location, highly accelerated rates were observed for several genes in *Pelargonium*, *Plantago*, and *Silene* (Zhu et al. 2016). Similar to legumes (Perry and Wolfe 2002), specific sequences appeared to give rise to the anomalous rates, for example, highly accelerated *rpoA* in *Pelargonium* had a rate more than 40 times higher than other IR genes. Increased taxon sampling included 22 species of *Pelargonium* and used a phylogenetic context to test rate heterogeneity relative to genomic location in specific lineages. Except for ribosomal protein genes, genes that were consistently located in the IR showed lower substitution rates than those in the SC regions in keeping with findings Perry and Wolfe (2002), and the hypothesis of Birky and Walsh (1992). However, among the 32 genes that showed clade specific variation of their genomic location the majority did not show significant rate changes relative to their position in the IR or SC regions. In *Pelargonium* and other taxa with atypical plastomes or highly accelerated sequences (i.e., *Silene*, *Plantago*), the observed heterogeneity could result from a mixture of locus-specific, lineage-specific and IR-dependent effects (Weng et al. 2017).

The novel IR in *M. minima* is adjacent to *ycf1* and includes *clpP*, two sequences that are often recognized as highly variable, possibly hotspots for recombination activity (Ruhlman and Jansen 2018). The hypothetical initiation of IR acquisition in the clade (figs. 1 and 6) involves a region that was found repeated five times in *Monsonia emarginata*, likely a recombinational hot spot in that species (Ruhlman et al. 2017). Rate and structural variation attributed to increased recombination activity was suggested for the IR-lacking legume *Lathyrus* (Magee et al. 2010) and *Plantago* (Zhu et al. 2016). Accumulation of sequence repeats likely plays a significant role in structural variation, and gene conversion during recombination between homologous/homeologous repeats can impact substitution rates. The decreased repeat content in *M. minima* relative to *M. suffruticosa* may be suggestive. A repeat-mediated phenomenon that originated in a common ancestor may have been brought to a halt in the *M*. *lupulina*/*M*. *minima* clade with the initiation of IR acquisition, marking another notable difference between IR gain in *Medicago* relative to *Erodium*. Despite reappearance of a large IR in *E. chrysanthum* repeats comprise more than 16% of its plastome (Blazier et al. 2016).

The relationship between repeat accumulation, IR loss or gain and plastome stability is unresolved while the influence of structural change, such as IR expansion, contraction or loss, on nucleotide substitution rates is somewhat less obscure. Given the limitations of previous hypotheses, it could be that that the IR is simply an artifact of plastome replication and lacks functional significance. It may be informative to investigate substitution rates in groups like *Erodium* and *Medicago* in much the same way others have looked at groups where genes have been included or excluded from the IR. Likewise denser sampling in the clade that includes *M. minima* may reveal variation in IR extent that could illuminate the specific mechanisms involved in IR acquisition. Unquestionably recombination underpins both conservation and variation in plastid genomes, however the precise mechanisms that govern plastome stability in taxa with or without the large IR remain to be elucidated.

## Supplementary Material

Supplementary data are available at *Genome Biology and Evolution* online.

## Supplementary Material

Supplementary Data

## Notes

**Data deposition:** Please note that detail of accession numbers is provided in supplementary table S1, supplementary material online.
