# Supplementary material for: Lost and Found: Return of the Inverted Repeat in the Legume Clade Defined by Its Absence
Source: Genome Biol Evol. 2019 Apr 8;11(4):1321–33. doi: 10.1093/gbe/evz076 (PMC6496590; doi:10.1093/gbe/evz076)
Supplement: Supplementary Data [file evz076_supp.docx]

**Supplementary figure legends**

Supplementary figure S1. **Alignment of the *Medicago* plastome *accD* coding region.** The predicted *accD* coding regions were extracted from newly completed plastomes of 19 *Medicago* species and *Trigonella*. *Medicago truncatula* was added to the dataset for comparison of conserved residues in the carboxy-terminus (Gurdon and Maliga 2014). Above, *accD* nucleotide alignment by translation (3870 nt, 64.3%); islands of conservation are indicated by brackets, size and pairwise nucleotide identity. The histogram above the alignment indicates percent identity: green, 100%; taupe, 30-99.9 %; red <30 %. Red line indicates conserved region (shown below) containing active residues. Below, thick black line indicates the 77 residue active site region. The entire protein was aligned with the Geneious aligner using default settings; percent amino acid identity is given below for the bracketed portion of the alignment. Red, putative acetyl-CoA binding site; blue: putative coA-carboxylation catalytic site; Green: carboxybiotin-binding site (Lee et al., 2004).

**Supplementary table S1.** Accession numbers and vouchers for newly sequenced taxa.

| Taxon | GRIN accession # | NCBI accession # | Voucher  accession # |
| --- | --- | --- | --- |
| *Trigonella foenum-graceum* | PI464822 | MK460508 | I.S. Choi MD025 |
| *Medicago radiata* | W6 37010 | MK460505 | I.S. Choi MD022 |
| *M. monspeliaca* | PI227051 | MK460506 | I.S. Choi MD023 |
| *M. biflora* | PI464827 | MK460504 | I.S. Choi MD019 |
| *M. suffruticosa* | PI516914 | MK460492 | I.S. Choi MD009 |
| *M. lupulina* | PI250937 | MK460497 | I.S. Choi MD015 |
| *M. minima* | PI641629 | MK460499 | I.S. Choi MD016 |
| *M. orbicularis* | PI253786 | MK460500 | I.S. Choi MD017 |
| *M. intertexta* | PI498831 | MK460501 | I.S. Choi MD021 |
| *M. laciniata* | PI141474 | MK460503 | I.S. Choi MD018 |
| *M. polymorpha* | PI250782 | MK460498 | I.S. Choi MD013 |
| *M.* x *blancheana* | PI495223 | MK460502 | I.S. Choi MD020 |
| *M. pironae* | PI253450 | MK460496 | I.S. Choi MD012 |
| *M. arborea* | PI504540 | MK460507 | I.S. Choi MD024 |
| *M. marina* | PI419391 | MK460495 | I.S. Choi MD011 |
| *M. cretacea* | PI631721 | MK460491 | I.S. Choi MD008 |
| *M. sativa* subsp. *glomerata* | PI632028 | MK460494 | I.S. Choi MD006 |
| *M. sativa* subsp. *sativa* | PI516588 | MK460489 | I.S. Choi MD003 |
| *M. sativa* subsp. *falcata* | PI499550 | MK460490 | I.S. Choi MD005 |
| *M. tetraprostrata* | PI577450 | MK460493 | I.S. Choi MD007 |

**Supplementary table S2:** The 69 genes shared across 28 taxa included in phylogenetic analysis.

*atpA*

*atpB*

*atpE*

*atpF*

*atpH*

*atpI*

*ndhA*

*ndhB*

*ndhC*

*ndhD*

*ndhE*

*ndhF*

*ndhG*

*ndhH*

*ndhI*

*ndhJ*

*ndhK*

*petA*

*petB*

*petD*

*petG*

*petL*

*petN*

*psaA*

*psaB*

*psaC*

*psaI*

*psaJ*

*psbA*

*psbB*

*psbC*

*psbD*

*psbE*

*psbF*

*psbH*

*psbI*

*psbJ*

*psbK*

*psbL*

*psbM*

*psbN*

*psbT*

*psbZ*

*rpl14*

*rpl16*

*rpl20*

*rpl32*

*rpl33*

*rpl36*

*rps2*

*rps3*

*rps4*

*rps7*

*rps8*

*rps11*

*rps12*

*rps14*

*rps15*

*rps19*

*rpoA*

*rpoB*

*rpoC1*

*rpoC2*

*ycf3*

*ccsA*

*cemA*

*clpP*

*matK*

*rbcL*

**Supplementary table S3.** Oligonucleotide primer sequences, target regions, expected results and NCBI accession numbers for PCR confirmation of repeats of interest and the *M. minima* inverted repeat.

| **CONFIRMATION OF REPEATS** | | |  |  |
| --- | --- | --- | --- | --- |
| Primer name | Sequence | |  |  |
| MSUF 1F | TTGAGGGAAACGCAGGACTG | |  |  |
| MSUF 1R | AGGGTTCCATCTCGTTGTGC | |  |  |
| MSUF 2F | GGCTACGTTCCATGCCTCAT | |  |  |
| MSUF 2R | GTCCCTTCGTCCATAACCCG | |  |  |
| MLUP 1F | CTCTATACACCGGAGCCCCT | |  |  |
| MLUP 1R | TGGTGAGCGTGGTTCGAAAT | |  |  |
| MLUP 2F | ACTCCGACAGCATCTAGGGT | |  |  |
| MLUP 2R | ACTTCCCGTTCGACTTGCAT | |  |  |
| MLUP 3F | GCCGATTGCTCTACCACTGA | |  |  |
| MLUP 3R | GTCCCTTCGTCCATAACCCG | |  |  |
| MLUP 4F | ACAAGAAACTACCGCAGAGG | |  |  |
| MLUP 4R | ACGAGTCGCACATACACCCTAG | |  |  |
| Primer pair | | Target species | Target region | kb |
| MSUF 01F-01R | | *M. suffruticosa* | clpP-rps12 5' intergenic spacer | 1 |
| MSUF 02F-02R | | *M. suffruticosa* | trnN(GUU)-ycf1 intergenic spacer | 1.2 |
| MLUP 01F-01R | | *M. lupulina* | rpl20-rps12 5'-clpP | 1.3 |
| MLUP 02F-02R | | *M. lupulina* | rps12 3'-trnV(GAC)-rrn16 | 1.5 |
| MLUP 03F-03R | | *M. lupulina* | trnN(GUU)-rps12 5'-ycf1 | 2.6 |
| MLUP 04F-04R | | *M. lupulina* | trnN(GUU)-rps12 5' intergenic spacer | 1.8 |

| **CONFIRMATION OF INVERTED REPEAT IN *M. MINIMA*** | | | | |
| --- | --- | --- | --- | --- |
| PrimerID | Primer sequence | Experimental  pairs | Target  region**^a^** | Expected size (accession #) |
| 1F | TATCCCGATGAGCCGAAACG | 01F-01R | LSC-IRA (a) | 888 bp  (MK490948) |
| 1R | GGGTGTATGTGCGACTCGTT | 02F-02R | IRA-SSC (b) | 1268 bp  (MK490949) |
| 2F | TCACGGTTTAGGCTGTTCCC | 03F-02F | SSC-IRB (c) | 1420 bp  (MK490954) |
| 2R | GTTGAGGTGGACGCACACTA | 01R-04R | IRB-LSC (d) | 948 bp  (MK490953) |
| 3F | TGGTTCAAGTCCAGGATGGC |  | | |
| 4R | ACCCGGTTCTTGCTGCTATT |  |  |  |

**^a^**lower case in bracket refers to Figure 2b (local map) and 2c (PCR result) in main text.
